# Supplementary material for: Microneedle-array patches loaded with dual mineralized protein/peptide particles for type 2 diabetes therapy
Source: Nat Commun. 2017 Nov 24;8:1777. doi: 10.1038/s41467-017-01764-1 (PMC5701150; doi:10.1038/s41467-017-01764-1)
Supplement: Supplementary file 1 — Supplementary Information [file 41467_2017_1764_MOESM1_ESM.pdf]

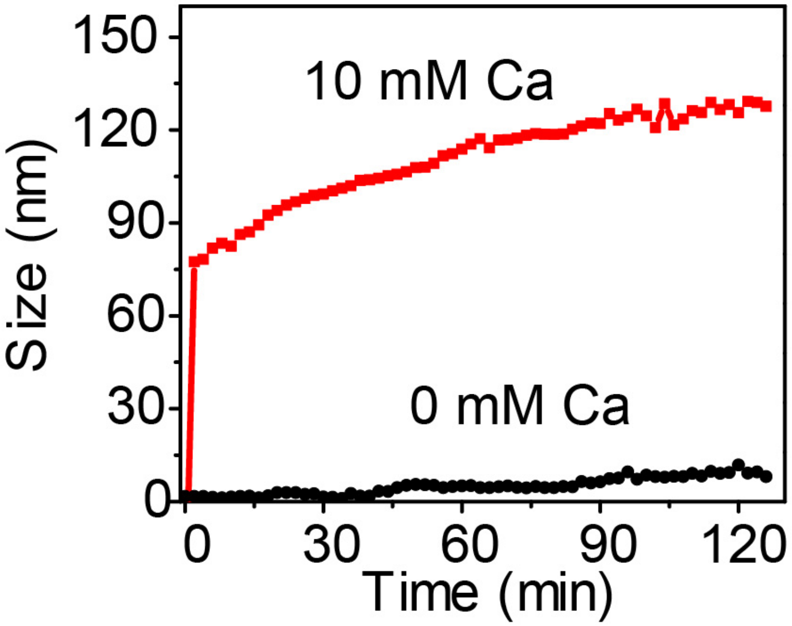

Supplementary Figure 1. Reaction kinetics of Ex4 mineralization after introducing calcium ions to the reaction medium.

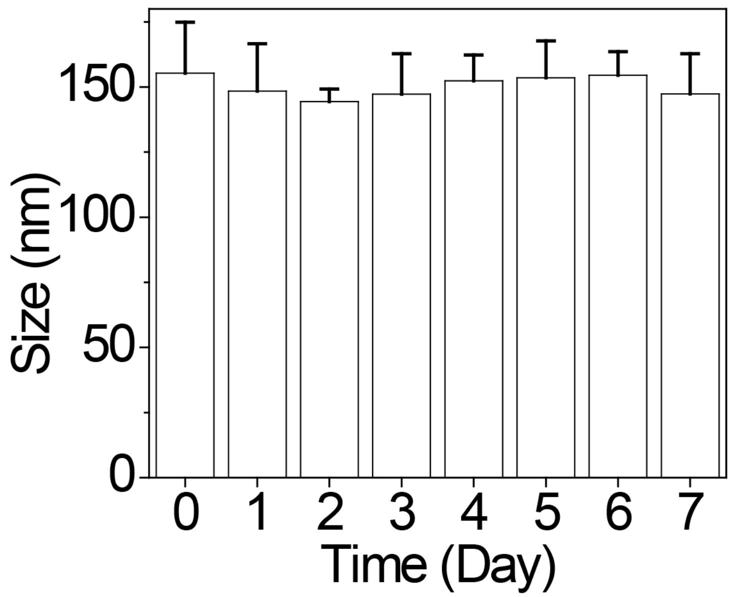

Supplementary Figure 2. Stability evaluation of m-Ex4 by size analysis at different time points. Mean ± S.D. (n = 3).

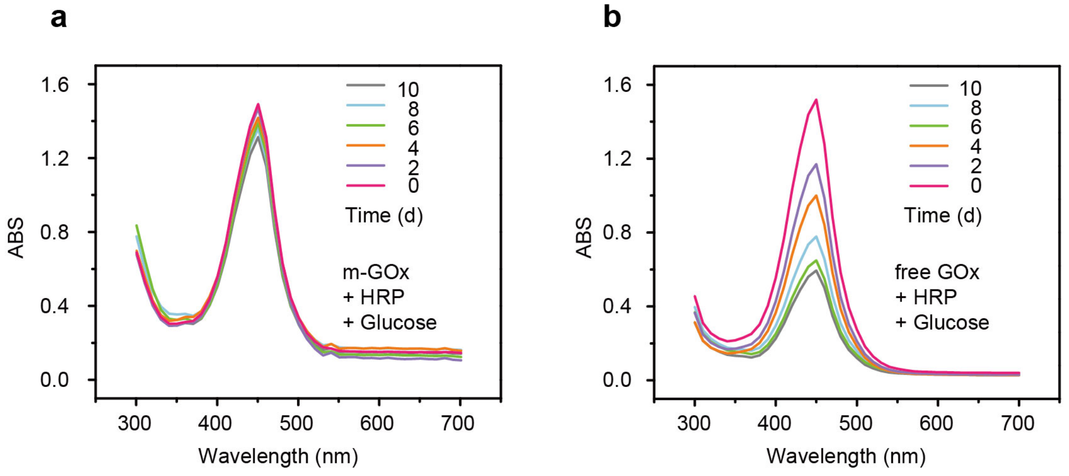

Supplementary Figure 3. UV-Vis absorption spectra of TMB solutions treated under different conditions. (a) m-GOx was stored at room temperature (25 °C) for a different time. (b) Free GOx was stored at room temperature (25 °C) for a different time.

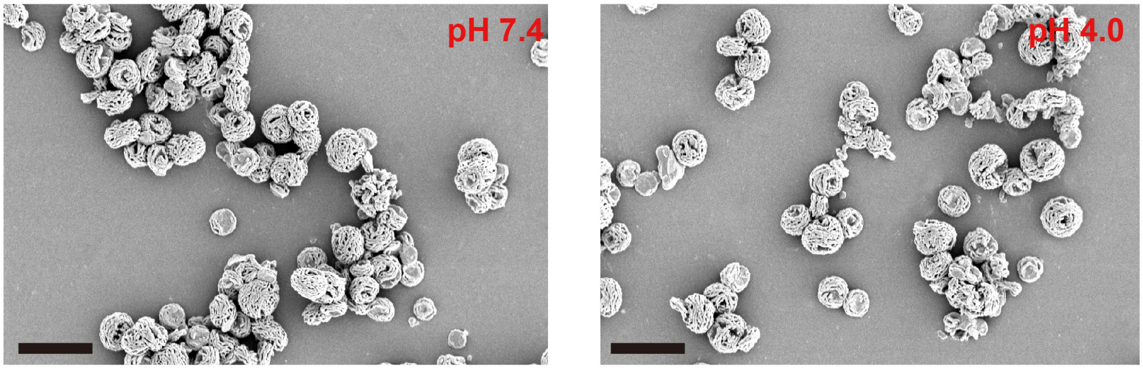

Supplementary Figure 4. SEM images of m-GOx at different pH values. Scale bar, 5 μm.

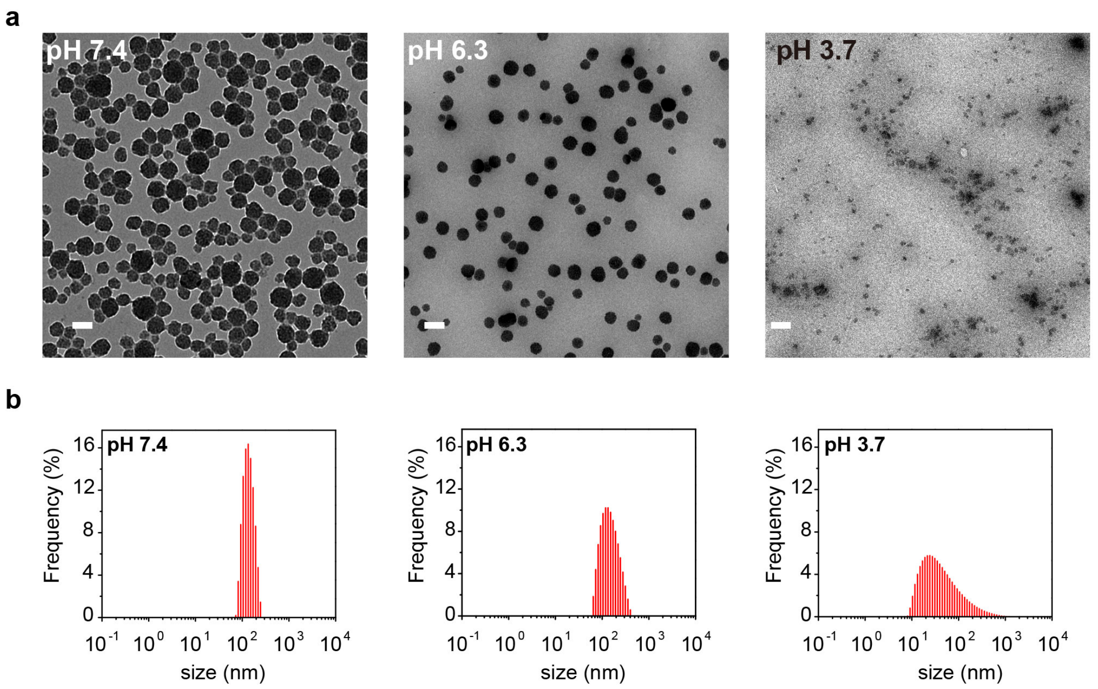

Supplementary Figure 5. Size change of m-Ex4 particles at different pH values. (a) TEM images of m-Ex4 particles at different pH values. Scale bar, 200 nm. (b) DLS analysis of m-Ex4 particles at different pH values.

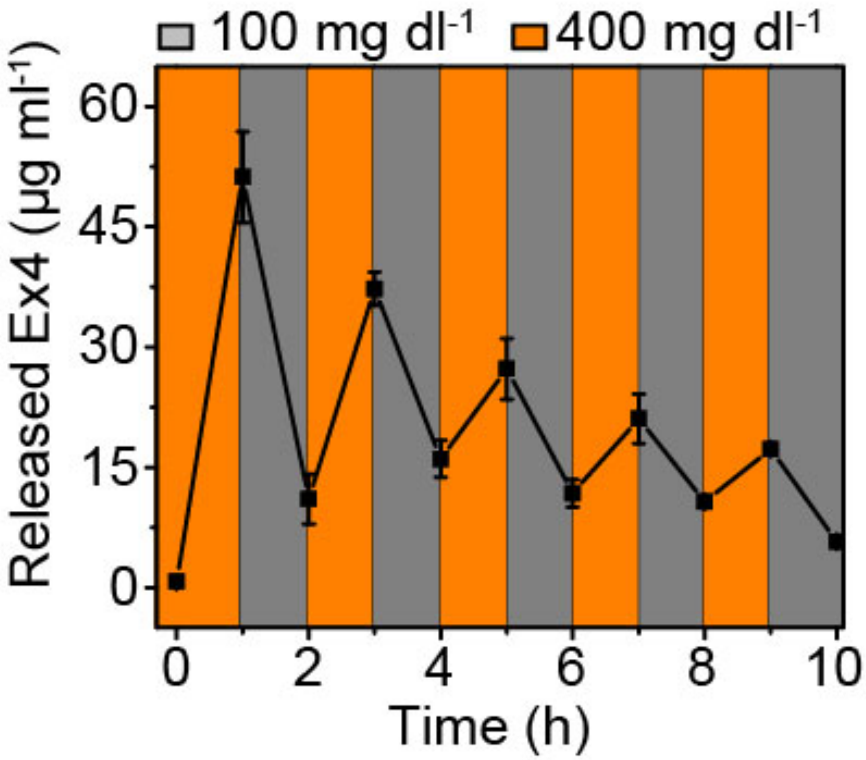

Supplementary Figure 6. The pulsatile release profile of alginate gel containing m-Ex4 and free GOx presented the concentration of released Ex4 as a function of glucose concentration. Mean ± S.D. (n = 3).

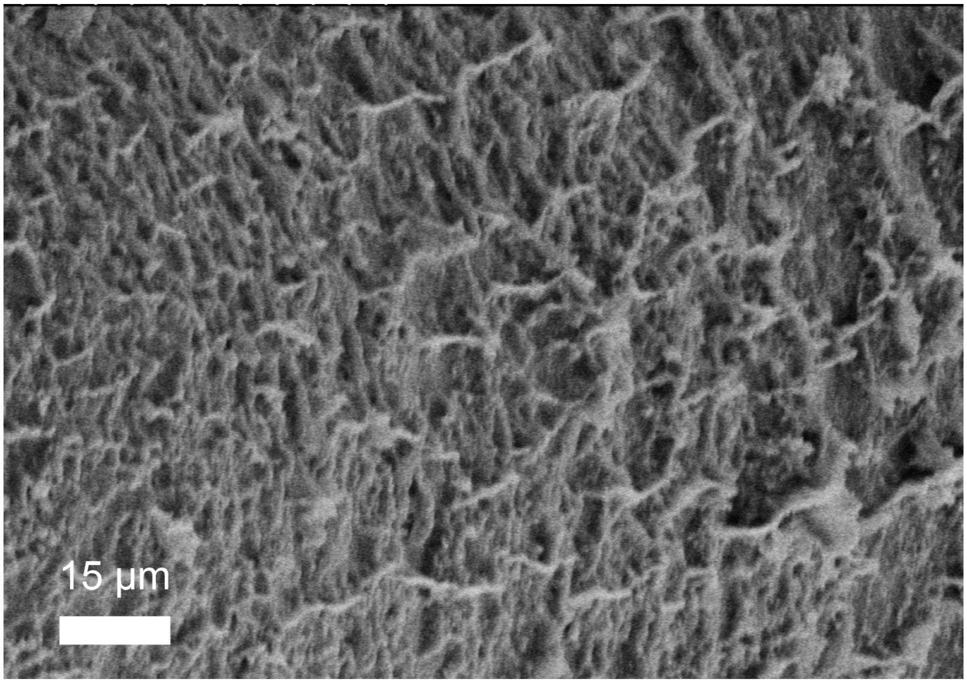

Supplementary Figure 7. SEM image of fabricated alginate hydrogel.

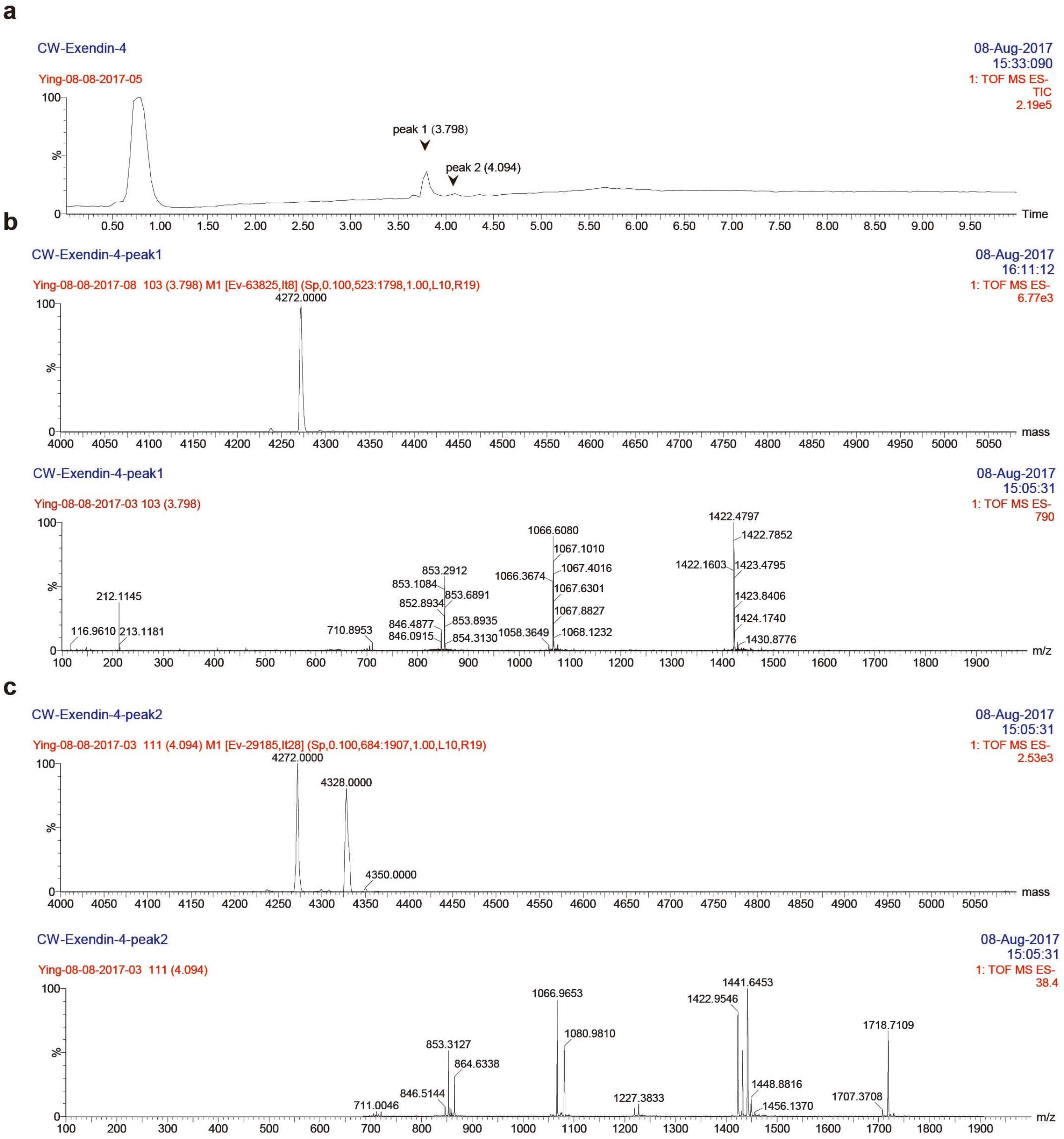

Supplementary Figure 8. Mass analysis of released Ex4. (a) HPLC indicates two peaks for released Ex4. (b) The main peak (peak1) shows the signal of 4272, which is corresponded to original Ex4 (The molecular weight of Ex4 is 4271). (c) Peak2 shows the m/s value of 4378 that is from the oxidation of –SH group in Cysteine to be –SOOH and sodium ion binding.

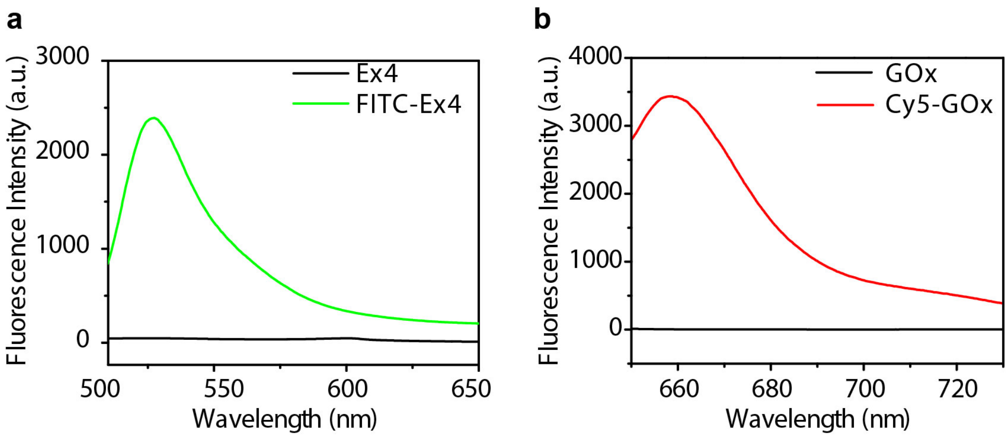

Supplementary Figure 9. The fluorescence emission spectra of dye-modified protein/peptide. (a) The fluorescence emission spectra of Ex4 and FITC-Ex4 under 495 nm excitation. (b) The fluorescence emission spectra of free GOx and Cy5-GOx under 646 nm excitation.

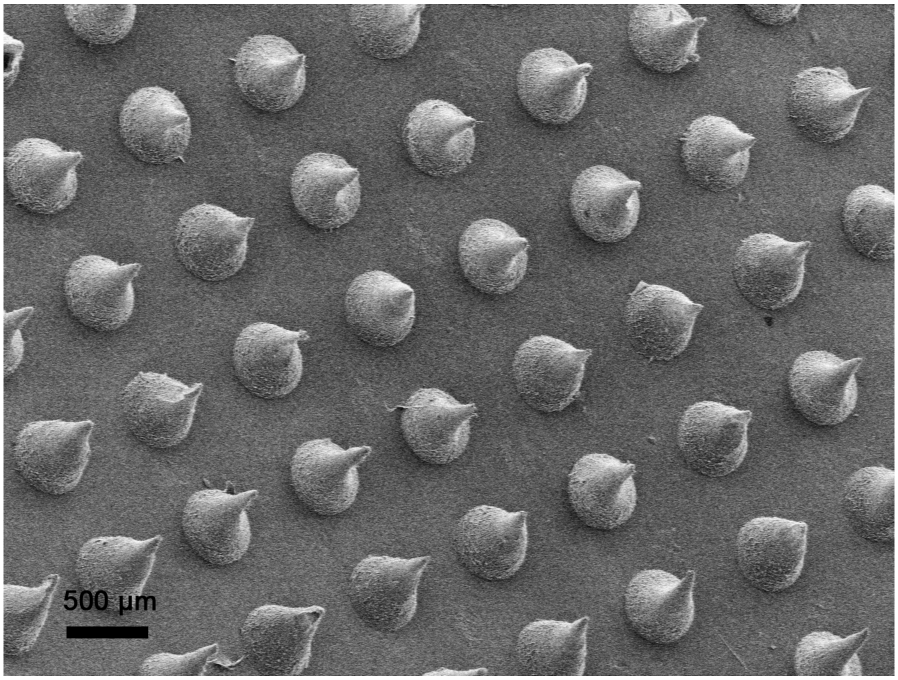

Supplementary Figure 10. SEM image of MN arrays after exposure to 400 mg ml^{-1} glucose solution for 24 h.

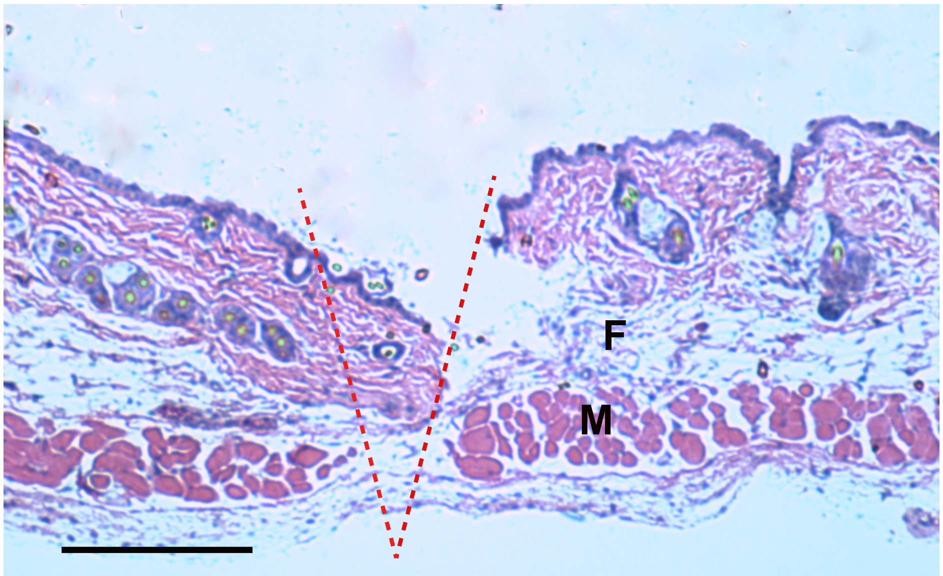

Supplementary Figure 11. Section of H&E stained mouse skin that was penetrated by a microneedle. The skin muscle and fat tissue regions are indicated by M and F, respectively. The region where MN insertion took place is indicated by the red dashed line. (Scale bar: 100 µm.)

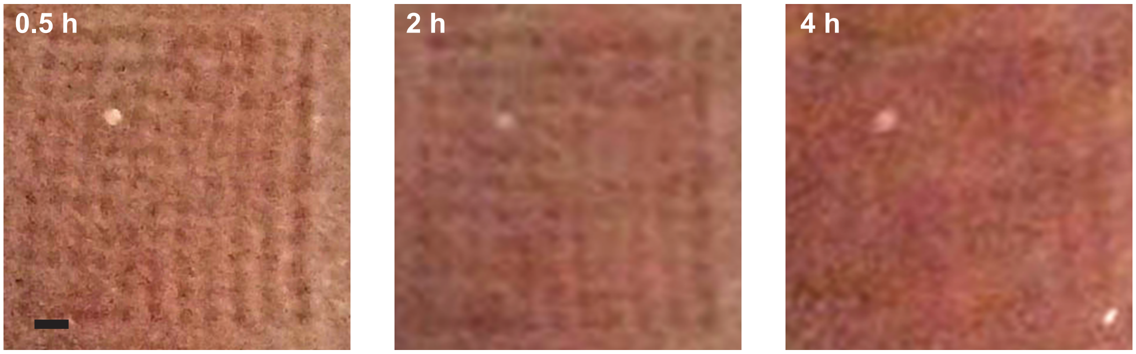

Supplementary Figure 12. Skin punctures at different time points. Scale bar, 250 µm. Clearly, the microchannels on the skin were created by insertion of the MN arrays. With an increase of post-administration time, the microchannels spontaneously disappeared and the surface of the skin is gradually recovered.

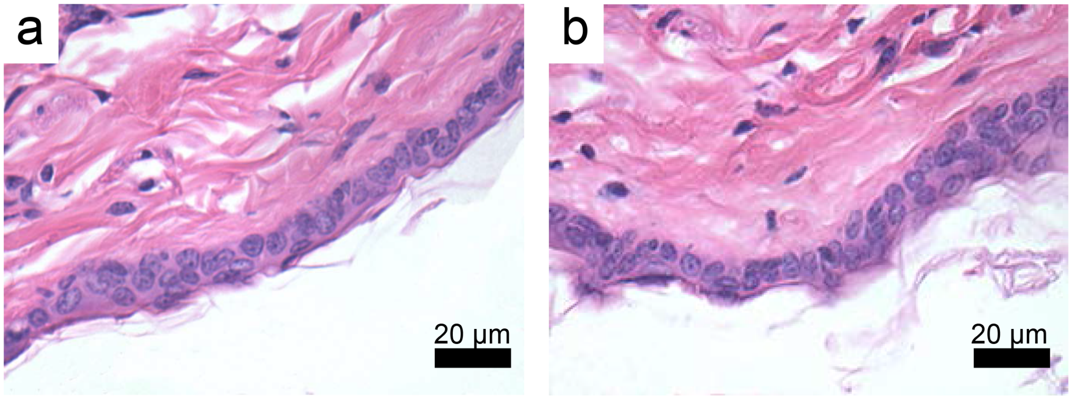

Supplementary Figure 13. H&E-stained skin sections for untreated and recovered samples. (a) Control skin without any MN administration. (b) Skin section upon 6 h recovery after MN administration.

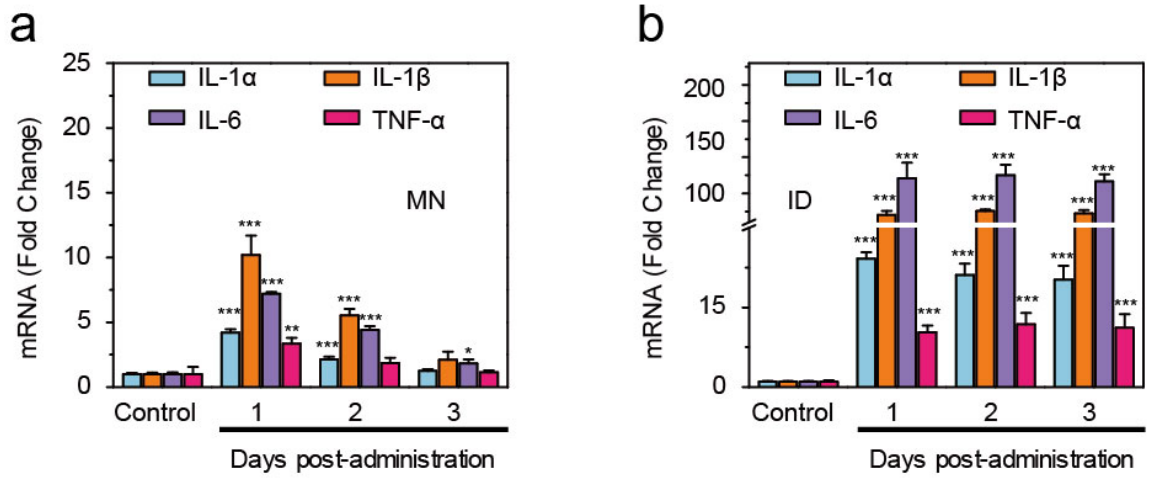

Supplementary Figure 14. Time-course effects of different treatments on proinflammatory cytokine gene expression in dermal tissues. (a) MN-array patch administration. (b) Intradermal (ID) injection. Obviously, MN-induced proinflammatory cytokine gene expression decreased much quicker than ID injection, probably due to the less invasiveness to the skin. Mean ± S.D. (n = 3). *$P < 0.05$, **$P < 0.01$, ***$P < 0.001$ compared with the control group (two-tailed Student's t-test).

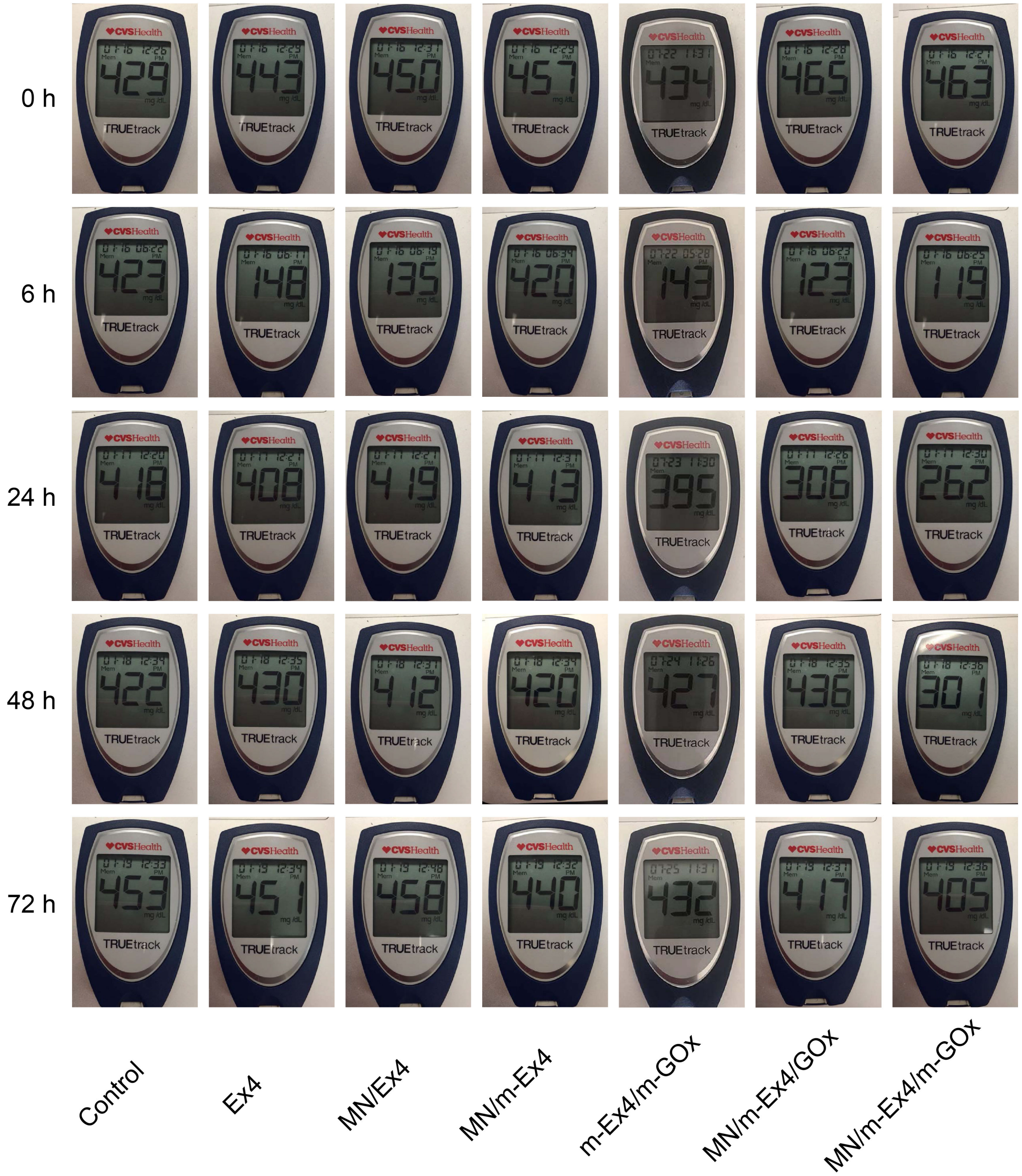

Supplementary Figure 15. Blood glucose level detection by one-touch glucose meter at different time points under various treatments.

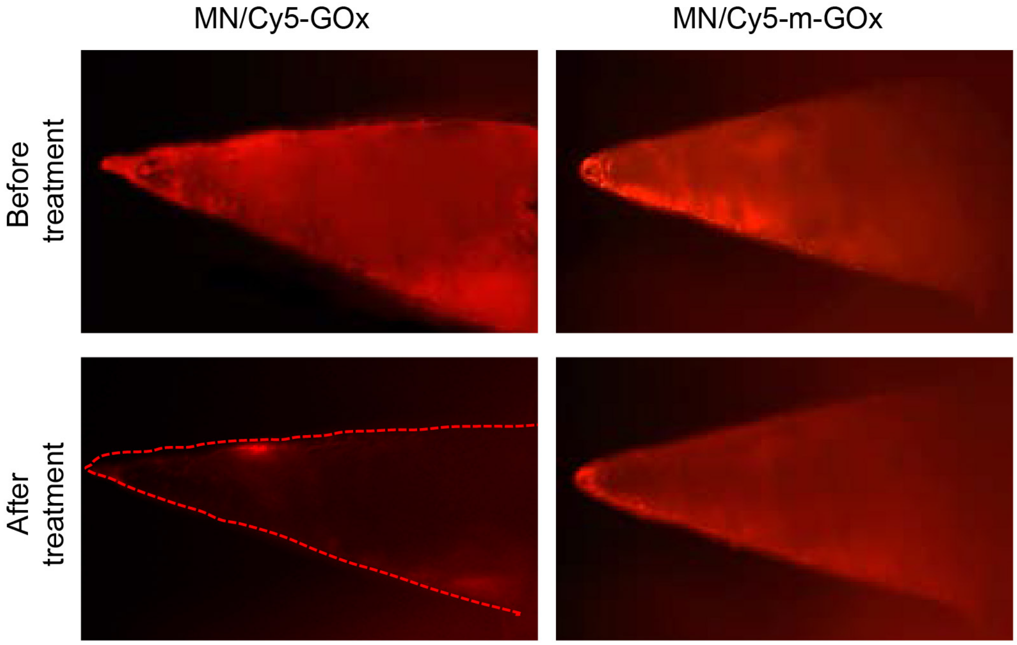

Supplementary Figure 16. Comparison of GOx leakage from MN/Cy5-GOx and MN/Cy5-m-GOx based on fluorescence observation.

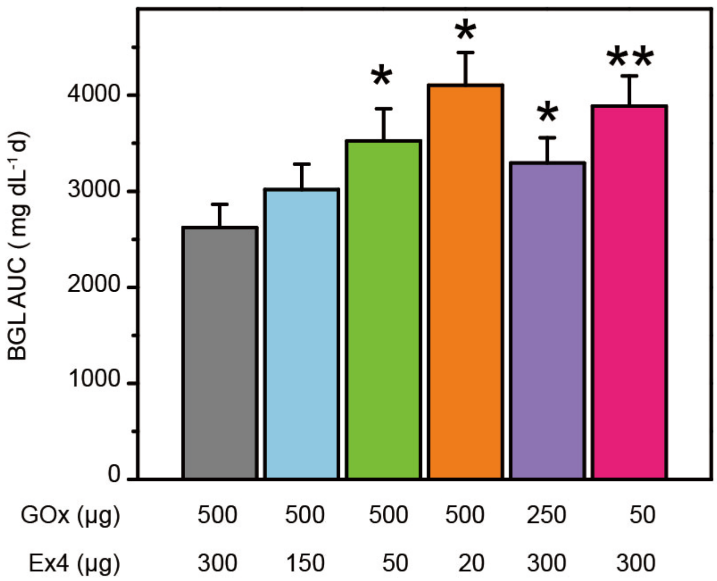

Supplementary Figure 17. The area under the curve (AUC) of BGL integrated up to 11 days under treatments of MN/m-Ex4/m-GOx patches which contain different Ex4 and GOx amounts. Mean ± S.D. (*n* = 3) **P* < 0.05, ***P* < 0.01 compared with the administration by the patch containing 500 μg GOx and 300 μg Ex4 (two-tailed Student's t-test).

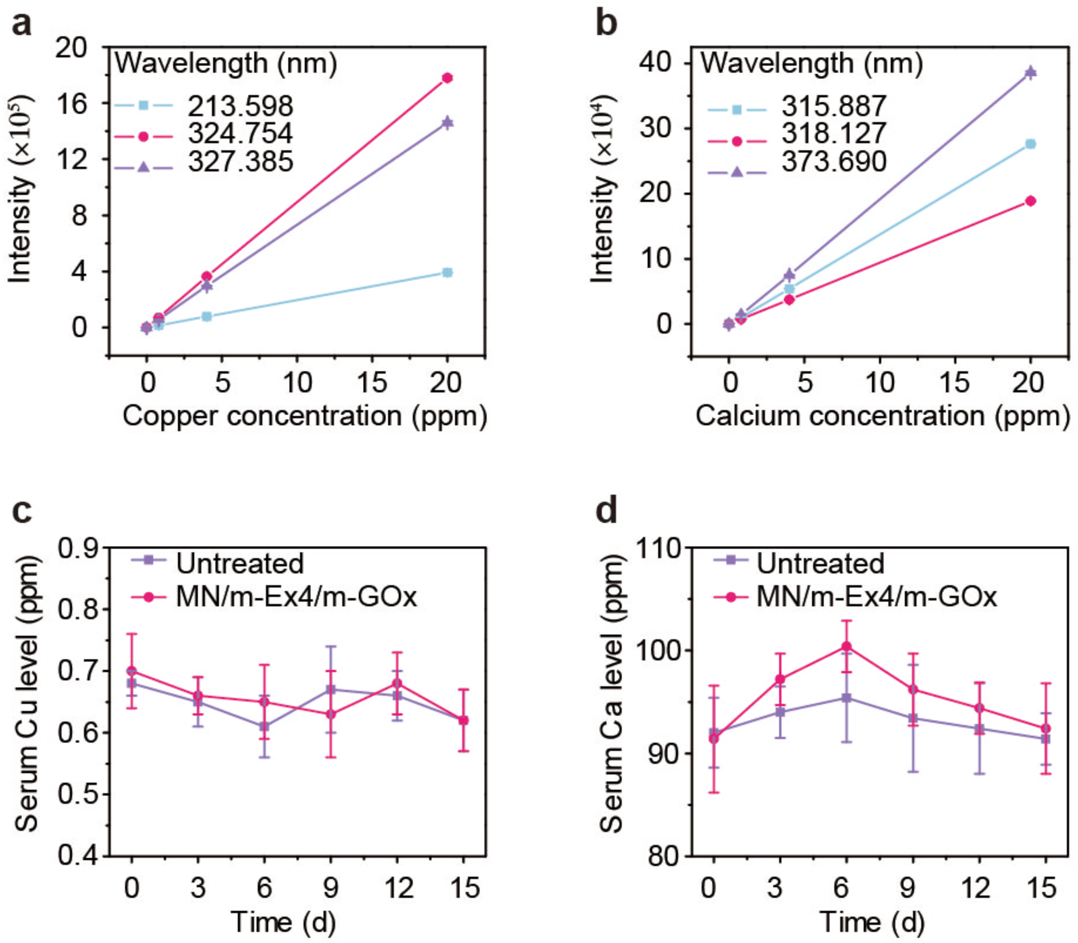

Supplementary Figure 18. Serum Cu and Ca detection by ICP-OES. Different wavelength all showed satisfactory linear relationship for copper (a) and calcium (b). (c) Serum Cu level had nearly no change during the treatment. Mean ± S.D. (n = 3). (d) Serum Ca level increased slightly and then dropped to a normal level during the treatment. Mean ± S.D. (n = 3).

Supplementary Methods

Materials

Cys40[leu14]-Ex4 was prepared by solid-phase peptide synthesis (CS Bio, Menlo Park, CA). The sequence of the peptide was confirmed to be His-Gly-Glu-Gly-Thr-Phe-Thr-Ser-Asp-Leu-Ser-Lys-Gln-leu-Glu-Glu-Glu-Ala-Val-Arg-Leu-Phe-Ile-Glu-Trp-Leu-Lys-Asn-Gly-Gly-Pro-Ser-Ser-Gly-Ala-Pro-Pro-Pro-Ser-Cys-NH_2 (molecular weight is around 4271). Alginate, Fluorescein isothiocyanate (FITC) and Glucose oxidase (GOx) were purchased from Sigma-Aldrich (USA). Cyanine5 (Cy5) NHS ester was bought from Lumiprobe (USA). Reaction medium, Dulbecco's Modified Eagle's Medium (DMEM), was obtained from Corning Incorporated (USA). All other chemicals were purchased from Fisher Scientific (USA) and used without further purification.

Characterization. Transmission electron microscopy (TEM) observations of m-Ex4 and m-GOx particles at different time points were performed on a Philips/FEI CM200 microscope (USA). Hitachi SU-70 Schottky field emission gun scanning electron microscopy (SEM) was applied for surface investigation and elemental mapping based on energy-dispersive X-ray spectroscopy (EDX). An SZ-100 Nanoparticle Analyzer (HORIBA Scientific, Japan) was used to measure the size distribution of particles up to one week. Fourier transform infrared (FTIR) spectroscopic analysis was performed on a Nicolet NEXUS 670 FTIR Spectrometer (GMI, USA). The thermogravimetric analysis (TGA) was performed using an SDT-Q600 (TA Instruments, USA) at a heating rate of 10 °C min^{-1} from 20 °C to 500 °C in a nitrogen atmosphere. The morphology of the MN patch was observed under the optical field by an Olympus BX41 microscope (Japan).

Ex4 and GOx labeling

For ease of observation, Ex4 and GOx were labeled by FITC and Cy5 dye, respectively**.** FITC was dissolved in dimethyl sulfoxide (DMSO, Sigma-Aldrich) at a final concentration of 2 mg ml^{-1}. Ex4 was dissolved in 0.1 M carbonate buffer (pH 9.0) at a final concentration of 2 mg ml^{-1}. For each 1 ml of protein solution, 50 μl of FITC solution

was very slowly added with gentle and continuous stirring of the protein solution. After all the required amounts of FITC solution were added, the reaction was incubated in the dark for 12 h at 4 °C. The unbound FITC was separated from the conjugate by PD-10 column (GE Healthcare). After freeze-drying, the FITC-Ex4 was obtained and stored at -20 °C before use. GOx was labeled by Cy5-NHS dye by a similar process. The fluorescence of FITC-Ex4 and Cy5-GOx was measured under an F-7000 fluorescence spectrophotometer (Hitachi, Tokyo, Japan).

GOx activity detection. The enzyme activity detection was based on a colorimetric reaction. Briefly, GOx or m-GOx (GOx dose was 0.5 mg) was added to 2 ml 100 mg dl^{-1} glucose for 25 min incubation at 37 °C. Then the supernatant was collected after centrifugation (10,000 rpm, 10 min). 100 µl of 10 mM tetramethylbenzidine (TMB), 20 µl of 10 µg ml^{-1} Horseradish peroxidase (HRP), and 1.72 ml of 0.2 M HAc-NaAC buffer (pH= 4.0) were added to the above 160 µl glucose reaction solution. The mixed solution was incubated at 37 °C for 20 min and then kept in an ice water bath for 10 min to terminate the reaction. After addition of 10 µl concentrated sulfuric acid, the absorbance of the final solution at 450 nm was measured on a Genesys 10S UV-Vis spectrophotometer (Thermo Scientific, USA), which indicated the catalytic activity of the enzyme[1].

Biocompatibility Analysis. After MN-array patch treatment and recovery for 6 hours, the mice were euthanized by isoflurane and the skin was excised. The tissues were fixed in 10% formalin and then embedded in paraffin, cut into 50-µm sections, and stained using hematoxylin and eosin for histological analysis.

In order to evaluate the safety of the MN-array patch, 50 µl blood samples were drawn from the orbital sinus at indicated time points (0, 3, 6, 9, 12, 15 days) during the patch treatments. For analysis of plasma samples, 20 µl samples were diluted by 1 ml of concentrated HNO_3 and boiled for 4 hours. The calcium and copper concentration in acidic solution were analyzed by 700 Series inductively coupled plasma optical emission spectrometry (ICP-OES, Agilent Technologies, USA), diluting with 3% HNO_3 if

necessary.

Evaluation of inflammatory response. The expressions of mRNAs for proinflammatory cytokines, IL-1α, IL-1β, IL-6 and TNF-α, in the dermal tissues were quantified by quantitative real-time polymerase chain reaction (qRT-PCR) after MN (MN/m-Ex4/m-GOx) or ID (m-Ex4/m-GOx) treatments. Total RNA was isolated from dermal tissues (approximately 30 mg) using RNAEasy kit (Qiagen, Germany), according to the manufacturer protocol. The extracted RNA was dissolved in 30 μl nuclease-free distilled water and stored at -20 °C. The concentration and purity of RNA were determined by Nanodrop Spectrophotometer (Thermo Scientific, USA). 300 ng obtained RNA was reversely transcripted into complementary DNA (cDNA) using Verso cDNA Synthesis Kit (Thermo Scientific, USA). Real-time PCR was performed using 1 μl template in a 20-μl reaction containing 0.5 μM of each primer and 10 μl Sybr Green Real-time PCR MasterMix (Applied Biosystems, USA). Each run consisted of 50 °C for 2 min and 95 °C for 10 min followed by 45 cycles of 95 °C for 15 s, 60 °C for 30 s, and 72 °C for 60 s in a 7300 real-time PCR system (Applied Biosystems, USA). GAPDH was used as a housekeeping gene for normalizing the expression data. The primer sequences are as follows:

IL-1α Forward: 5'-CCGAGTTTCATTGCCTCTTT-3'
IL-1α Reverse: 5'-ACTGTGGGAGTGGAGTGCTT-3'

IL-1β Forward: 5'-CACCTTCTTTTCCTTCATCTTTG-3'
IL-1β Reverse: 5'-GTCGTTGCTTGTCTCTCCTTGTA-3'

IL-6 Forward: 5'-TGATGGATGCTTCCAAACTG-3'
IL-6 Reverse: 5'-GAGCATTGGAAGTTGGGGTA-3'

TNF-α Forward: 5'-ACTGAACTTCGGGGTGATTG-3'
TNF-α Reverse: 5'-GCTTGGTGGTTTGCTACGAC-3'

GAPDH Forward: 5’-GGTCTCCTCTGACTTCAACA-3’

GAPDH Reverse: 5'-AGCCAAATTCGTTGTCATAC-3'

Supplementary References

1 Sun, J. *et al.* Multi-enzyme co-embedded organic–inorganic hybrid nanoflowers: synthesis and application as a colorimetric sensor. *Nanoscale* **6**, 255-262 (2014).
